# The Association between Sleep and Bone Mineral Density: Cross‐Sectional Study Using Health Check‐up Data in a Local Hospital in Japan

**DOI:** 10.1002/jbm4.10820

**Published:** 2023-09-30

**Authors:** Reiko Yamaura, Hideko Kasahara, Satoshi Iimuro, Tsutomu Yamazaki

**Affiliations:** ^1^ Graduate School of Medicine International University of Health and Welfare Tokyo Japan; ^2^ International University of Health and Welfare School of Medicine Narita Japan; ^3^ Innovation and Research Support Center International University of Health and Welfare Tokyo Japan

**Keywords:** SLEEP DURATION, INSOMNIA, BONE MINERAL DENSITY (BMD), OSTEOPOROSIS, JAPANESE, POSTMENOPAUSAL WOMEN

## Abstract

This study aimed to investigate the association between daily sleep duration of <7 hours and lower bone mineral density (BMD) using data from annual health check‐ups conducted in Japan between 2020 and 2022. Multivariate regression models were used, where BMD was the objective variable and daily sleep duration (<5 hours, 5 to <7 hours, 7 to <9 hours [reference], ≥9 hours) was the exposure variable adjusted for age, body mass index, physical activity, smoking status, and alcohol intake for men and women and further adjusted for menopausal status for women. The association between insomnia and BMD was also investigated. BMD was determined using calcaneal quantitative ultrasound and expressed as a percentage of the young adult mean (%YAM). In total, 896 men and 821 women were included. Median age was 54 years (interquartile range [IQR]: 46 to 64) for men and 55 years (IQR: 46 to 64) for women). Median BMD for men and women was 79%YAM (IQR: 71 to 89) and 75%YAM (IQR: 68 to 84), respectively. Approximately 80% of men and women slept <7 hours daily. Multivariate regression showed no association between sleep duration and BMD in men. However, women who slept 5 to <7 hours daily had significantly higher BMD by 3.9% compared with those who slept 7 to<9 hours (*p* = 0.004). No association between insomnia and BMD was found. Overall, a daily sleep duration of <7 hours was not independently associated with lower BMD compared to those who slept 7 to <9 hours in men and women. However, as there is evidence of both shorter and longer sleep durations being associated with an increased risk of adverse events, including cardiovascular events, our result needs to be interpreted with caution. © 2023 The Authors. *JBMR Plus* published by Wiley Periodicals LLC on behalf of American Society for Bone and Mineral Research.

## Introduction

Osteoporosis has been a common public health problem worldwide, and its prevalence is on the rise.^[^ ^1^ ^]^ In Japan, which has a rapidly aging population, 10% of the total population have osteoporosis, especially women ≥70 years old (approximately 40%).^[^ ^2^ ^]^ Such a high prevalence has detrimental effects on the health of older individuals, resulting in increased healthcare costs^[^ ^3^ ^]^ and caregiver burden.^[^ ^4^ ^]^ Therefore, it is crucial to prioritize the preservation of bone health and implement preventive measures against osteoporotic fractures as an urgent public health priority.

Bone health is affected by a combination of nonmodifiable and modifiable risk factors. Nonmodifiable factors, such as sex, age, and hormone levels, and menopausal status play a significant role.^[^ ^5^ ^]^ Additionally, several modifiable risk factors can contribute to poor bone health, including low body mass index (BMI),^[^ ^6^ ^]^ smoking,^[^ ^7^ ^]^ excessive alcohol consumption,^[^ ^8^ ^]^ and physical inactivity.^[^ ^9^ ^]^ It is worth noting that metabolic syndromes like diabetes mellitus (DM)^[^ ^10^ ^]^ and dyslipidemia^[^ ^11^ ^]^ can also lead to a decrease in bone mineral density (BMD) due to the presence of oxidative stress.

Emerging research suggests that inadequate sleep may contribute to poor bone health and represents a modifiable risk factor. Swanson et al. reported that the circadian system affects bone health through the imbalance between born resorption and formation.^[^ ^12^ ^]^ Furthermore, several studies conducted in the United Kingdom (UK), the United States, and China demonstrated an association between shorter daily sleep durations (less than 5, 6, and 7 hours) and lower BMD or an increased risk of osteoporosis.^[^ ^13^, ^14^, ^15^, ^16^ ^]^ Notably, the recommended sleep durations provided by the US National Sleep Foundation are 7 to 9 hours for adults aged 26 to 64 years and 7 to 8 hours for individuals aged 65 years and older.^[^ ^17^ ^]^

In Japan, a significant proportion of adults, ranging from 70% to 80%, reported sleeping less than 7 hours per day.^[^ ^18^ ^]^ However, the impact of this sleep duration on decreased BMD remains unclear. To date, Kobayashi et al. reported longer daily sleep duration (≥8 hours, reference <6 hours) was associated with higher odds of osteoporosis,^[^ ^19^ ^]^ whereas Sasaki et al. found no association between sleep duration and BMD using sleep duration as a continuous variable.^[^ ^20^ ^]^ However, neither of these studies accounted for the U‐shaped relationship between daily sleep duration and BMD, where both shorter and longer sleep durations were found to be associated with decreased BMD in other research studies.^[^ ^14^, ^21^ ^]^

Therefore, this study aimed to investigate the relationship between sleep duration, sleep quality, and BMD using data from annual health check‐ups conducted at a local hospital in Japan.

## Methods

### Settings and study participants

This study employed a cross‐sectional study design. All data of the participants were collected at the time of an annual health check‐up at the Center for Preventive Medicine of the International University of Health and Welfare Narita Hospital in Narita City (approximately 50 km east of Tokyo), Japan, between May 2020 and July 2022. The center has been offering health check‐ups primarily for city residents or workers since May 2020. In Japan, annual health check‐ups aim to detect chronic diseases and identify associated risk factors at an early stage. The costs of these health check‐ups are subsidized by the government and employers. Among the participants who underwent the health check‐up, approximately 10% received BMD tests either because the tests were included as part of the supported health check‐up program or because the participants expressed interest in undergoing the tests. To be included in this study, participants had to be 20 years of age or older. In cases where participants underwent multiple health check‐ups during the study period, only the results from their first set of tests were considered for analysis.

The annual health check‐up program relevant to this study consisted of a pre‐examination questionnaire and initial evaluations (e.g., vital signs and laboratory data). The pre‐examination questionnaire contained self‐reported lifestyle information (e.g., exercise, sleep, diet, and working status) and clinical information (e.g., current comorbidities and medications, clinical history, and family clinical history). Demographic information (excluding socioeconomic and educational information) was retrieved from health insurance information.

Under the Japanese ethics guidelines governing research using existing data, the need to obtain informed consent was waived. Approval for this study was granted by the Institutional Review Board of the International University of Healthcare and Welfare (permission number: 22‐Im‐004).

### Outcome assessment

BMD was measured using a quantitative ultrasound system. Each test was performed with a CM‐300 (Furuno Electric Co., Ltd., Nishinomiya, Hyogo, Japan) on the right calcaneus by certified technicians. BMD was derived from the percentage of young adult mean (%YAM), which is the relative percentage of current BMD when the average BMD for those aged 20 to 44 years by sex was set at 100%. If an individual's %YAM was ≤70%, the person would be recommended to visit an orthopedist to undergo a full examination using dual‐energy X‐ray absorptiometry for a definitive diagnosis of osteoporosis.

### Exposure assessment

Exposure valuables included daily sleep duration (<5 hours, 5 to <7 h, 7 to <9 hours, or ≥9 hours) and insomnia. Sleep duration and insomnia were assessed solely based on responses to the pre‐examination questionnaire. For the evaluation of insomnia, participants were considered to have insomnia if they met the following two conditions: (1) participants who indicated “nonrestorative sleep” in response to the question “Do you get enough rest with sleep?” (2) participants who reported one or more of the following insomnia symptoms: “difficulty maintaining sleep,” “difficulty initiating sleep,” “early‐morning awakening,” and “taking sleep medication.” These symptoms were defined based on the criteria outlined in the *International Classification of Sleep Disorders*, Third Edition.^[^ ^22^ ^]^ The sleep‐related questions in the pre‐examination questionnaire were designed to capture participants' subjective sleep health in a general sense. No specific duration or scale was provided. The details of the questionnaire, including the specific wording of the questions, can be found in Table S1, which provides additional information for reference.

### Covariate assessment

Covariates used in this study were chosen based on current Japanese osteoporosis guidelines^[^ ^23^ ^]^ and the relevant literature. The covariates were age (continuous), sex (men or women), BMI (continuous),^[^ ^6^ ^]^ fracture history (yes or no), early menopause (early or not early),^[^ ^24^ ^]^ physical activity (active or not),^[^ ^9^ ^]^ smoking (current, ex‐smoker, or never),^[^ ^7^ ^]^ alcohol intake (excessive drinker or not),^[^ ^8^ ^]^ causative diseases that induce oxidative stress such as DM^[^ ^10^ ^]^ (yes or no) and DL^[^ ^11^ ^]^ (yes or no), use of sleep medication (yes or no), use of osteoporosis medication (yes or no), and working pattern (daytime worker, shift worker, or night‐shift worker).

Information on age and sex was obtained from the participants' medical insurance records. BMI was derived from measurements taken during the initial evaluation. Fracture history, excluding traumatic fractures, was assessed using data provided in the pre‐examination questionnaire. Menopause was reported in the pre‐examination questionnaire; early menopause in this study was defined as 45 years or younger.^[^ ^25^ ^]^

Individuals were considered to be physically active if they exercised for at least 30 minutes per session, two or more times per week. Excessive drinker was defined as an individual who consumed two or more alcoholic drinks (i.e., containing ≥20 g ethanol; for example, 500 mL of beer is equal to one drink).^[^ ^23^ ^]^ DM and DL were assessed based on either laboratory data or the pre‐examination questionnaire. If laboratory values of HbA1C were ≥6.5%, the case would be treated as DM.^[^ ^26^ ^]^ Otherwise, if LDL‐C ≥ 140 mg/dL, HDL‐C < 40 mg/dL, or non‐HDL‐C ≥ 170 mg/dL, it would be treated as DL.^[^ ^27^ ^]^ In addition, participants who reported visiting or receiving ongoing treatment, having a medical history, or indicating past medical conditions in the pre‐examination questionnaire were classified as having a medical condition. Similarly, participants who indicated being at least 65 years old and did not specify any particular working pattern were categorized as daytime workers.

Osteoporosis, fracture history, and working pattern were described but not included in the models assessing the association between sleep duration, insomnia, and BMD. This decision was made due to the small proportion of participants reporting these variables, despite their known association with refracture risk^[^ ^28^ ^]^ and osteoporosis.^[^ ^29^ ^]^ Additionally, participants who used sleep or osteoporosis medication were excluded from the analysis to minimize potential confounding effects on the outcomes of interest. However, other covariates were included in the models for adjusting and conducting subgroup analyses.

### Statistical analysis

All descriptive summaries were computed as *n* (%) or median (interquartile range [IQR]) for categorical and continuous variables, respectively. To assess the association between sleep duration or insomnia with BMD, we performed multivariate regression analyses using subset data of each sex. We created three models: model 1 adjusted for age, menopausal status, early menopause, and BMI (menopausal status was used only for women and early menopause was used only for postmenopausal women); model 2, which was further adjusted for physical activity, smoking, and alcohol intake; and model 3, which was further adjusted for causative diseases (DM and DL). We checked collinearity among the covariates, but no strong correlation was found (−0.8 > *r* > 0.8).

To examine the effect of menstruation, subgroup analysis was conducted for pre‐ and postmenopausal women. The results were assessed by a normal Q‐Q plot, and BMD was logarithmically transformed to obtain a more normal distribution. We computed the Akaike information criterion (AIC) to see model fitness because DM and DL were not well‐established risk factors compared to other variables selected in model 2. If model 3 showed higher value in AIC than model 2, then model 3 was considered. To address the potential impact of missing values, multiple imputation methods were employed for records with a missing rate of 5% or higher within each analysis set.^[^ ^28^ ^]^ All statistical analyses were conducted using R software version 4.2.1 or higher (R Foundation for Statistical Computing, Vienna, Austria), and a two‐tailed level of *p*‐value <0.05 was regarded as statistically significant and results were presented 95% confidence interval (CI).

## Results

### Participants

Participant backgrounds are summarized in Table 1. Men (*N* = 896) and women (*N* = 821) received BMD tests, with 63.5% of the women being postmenopausal (*N* = 521). The median age and BMI were 54 years (IQR: 46 to 64) and 24.4 kg/m^2^ (IQR: 22.3 to 26.7) in men, respectively, and 55 years (IQR: 46 to 64) and 21.9 kg/m^2^ (IQR: 19.7 to 24.3) in women, respectively (43 years [IQR: 39 to 48] and 21.4 kg/m^2^ [IQR: 19.4 to 23.6] in premenopausal women, respectively, and 61 years [IQR: 56 to 67] and 22.2 kg/m^2^ [IQR: 20.2 to 24.6] in postmenopausal women, respectively). Approximately 30% of men and 25% women were physically active (15% and 30% of pre‐ and postmenopausal women, respectively) and 23% were never smokers among men and 72% among women (69% and 74% in pre‐ and postmenopausal women, respectively). More than 90% of participants were daytime workers.

**Table 1:** Characteristics of Participants by Sex and Menopausal Status

	Men (*N* = 896)	Women (*N* = 821)	Premenopausal women (*N* = 273)	Postmenopausal women (*N* = 521)
Age, years	54 (46, 64)	55 (46, 64)	43 (39, 48)	61 (56, 67)
BMI, kg/m^2^	24.4 (22.3, 26.7)	21.9 (19.7, 24.3)	21.4 (19.4, 23.6)	22.2 (20.2, 24.6)
Missing	0	7	6	1
Postmenopausal women	–	521 (66%)	–	–
Missing		26		
Early menopause, *n* (%)	–	63 (8.4%)	–	63 (13.1%)
Missing		68		41
Physically active, *n* (%)	270 (30.2%)	201 (24.7%)	41 (15.0%)	156 (30.0%)
Missing	2	6	0	1
Smoking status, *n* (%)				
Current smoker	272 (30.4%)	64 (7.8%)	24 (8.8%)	34 (6.6%)
Past smoker	416 (46.6%)	167 (20.4%)	60 (22.0%)	102 (19.7%)
Never smoker	205 (23.0%)	586 (71.7%)	189 (69.2%)	383 (73.8%)
Missing	3	4	0	2
Excessive drinker, *n* (%)	95 (10.7%)	14 (1.7%)	5 (1.8%)	9 (1.7%)
Missing	4	4		2
Bone mineral density, %YAM	79 (71, 89)	75 (68, 84)	81 (75, 91)	71 (65, 79)
Osteoporosis	3 (0.3%)	58 (7.1%)	2 (0.7%)	56 (10.7%)
History of fracture, *n* (%)	3 (0.3%)	21 (2.6%)	1 (0.4%)	20 (3.8%)
Osteoporotic medicine use, *n* (% to history of fracture)	2 (66.7%)	19 (90.5%)	1 (100%)	18 (90.0%)
Dyslipidemia, *n* (%)	541 (60.4%)	405 (49.3%)	76 (27.8%)	325 (62.4%)
Diabetes mellitus, *n* (%)	162 (18.1%)	55 (6.7%)	6 (2.2%)	48 (9.2%)
Work style, *n* (%)				
Daytime worker	779 (93.3%)	679 (94.8%)	237 (92.9%)	431 (96.2%)
Shift worker	55 (6.6%)	32 (4.5%)	17 (6.7%)	13 (2.9%)
Night worker	1 (0.1%)	5 (0.7%)	1 (0.4%)	4 (0.9%)
Missing	61	105	18	73

*Note*: Continuous variables are presented as median in first and third quartiles, and categorical variables are presented as a percentage of total excluding missing.

Abbreviations: BMI = body mass index; YAM = young adult mean.

### BMD and osteoporotic fracture

Table 1 includes BMD and osteoporotic fracture status. Median BMD in men and women was 79%YAM (IQR: 71 to 89) and 75%YAM (IQR: 68 to 84), respectively, and was higher in premenopausal women compared with in postmenopausal women (81%YAM [IQR: 75 to 91] versus 71%YAM [IQR: 65 to 79]).

Self‐reported osteoporosis was 0.3% and 7.1% in men and women, respectively, with 0.7% and 10.7% in pre‐ and postmenopausal women, respectively. Also, among participants who reported osteoporosis, 66.7% of men and 90.5% of women (100.0% and 90.0% in pre‐and postmenopausal women) used osteoporosis medication.

### Sleep duration and insomnia

Self‐reported daily sleep duration and sleep satisfaction are summarized in Table 2. Seventy percent to 74% of participants in each group slept 5 to <7 hour followed by 7 to <9 hours (approximately 20%) daily. Only a small proportion (0 to 0.6% in each group) of participants reported ≥9 hours of sleep. Overall, approximately 80% of participants slept <7 hours. Participant characteristics by sleep duration are presented in Table S2. Although less than 10% of participants reported <5 hours sleep, their median BMI was higher at 25.8 kg/m^2^ (IQR: 23.1 to 28.4), 23.2 kg/m^2^ (IQR: 21.5 to 25.8), and 24.1 kg/m^2^ (IQR: 21.7 to 26.0) in men, women, and postmenopausal women, respectively, compared to participants who reported longer sleep durations (5 to <7 hour, 7 to <9 hours, and ≥9 hours), but not in premenopausal women. In contrast, median BMI in those who reported ≥9 hours of sleep was lower in men, women, and postmenopausal women.

**Table 2:** Self‐reported Daily Sleep Duration and Sleep Satisfaction by Sex and Menopausal Status

	Men (*N* = 896)	Women (*N* = 821)	Premenopausal women (*N* = 273)	Postmenopausal women (*N* = 521)
Sleep duration, *n* (%)				
<5 hours	73 (8.2%)	58 (7.2%)	17 (6.3%)	37 (7.2%)
5 to <7 hours	629 (70.4%)	592 (73.2%)	200 (74.1%)	376 (72.7%)
7 to <9 hours	189 (21.1%)	156 (19.3%)	53 (19.6%)	101 (19.5%)
≥9 hours	3 (0.3%)	3 (0.4%)	0 (0.0%)	3 (0.6%)
Missing	2	12	3	4
Sleep satisfaction, *n* (%)				
Sleep well	361 (41.4%)	327 (42.4%)	134 (49.4%)	189 (38.8%)
Difficulty in maintaining sleep	266 (30.5%)	283 (36.7%)	95 (35.1%)	180 (37.0%)
Difficulty in initiating sleep	114 (13.1%)	89 (11.5%)	29 (10.7%)	58 (11.9%)
Early‐morning awaking	81 (9.3%)	37 (4.8%)	8 (3.0%)	29 (6.0%)
Taking sleep medication	22 (2.5%)	17 (2.2%)	3 (1.1%)	14 (2.9%)
Multiple answers^a^	28 (3.2%)	19 (2.5%)	2 (0.7%)	17 (3.5%)
Missing	24	49	2	34
Not enough rest with sleep, *n* (%)	325 (36.9%)	333 (42.9%)	101 (37.0%)	222 (45.3%)
Missing	16	44	0	31
Insomnia, *n* (%)	261 (29.9%)	257 (33.3%)	69 (25.5%)	180 (37.0%)
Missing	24	49	2	34

*Note*: Continuous variables are presented for median with first and third quartiles, and categorical variables are presented for % to the total (denominator excludes missing counts).

^a^ Individuals who did not select “Sleep well” and selected ≥2 answers from others (Difficulty in maintaining sleep, Difficulty in initiating sleep, Early‐morning awaking, or Taking sleep medication) were counted as “multiple answers”. Of participants who were included in multiple answers, each *n* = 3, 5, 1, 4 for men, women, premenopausal women, postmenopausal women who reported “Taking sleep medication” were excluded from the univariate and multivariate regression analyses.

While approximately 40% of men and women reported sleeping well (more premenopausal women than postmenopausal women: 49% versus 39%), more than 30% of participants reported difficulty in maintaining sleep, followed by difficulty in initiating sleep (≥10%). More than 2% of participants took sleep medication (in women, there were more postmenopausal women than premenopausal women: 2.9% versus 1.1%). Thirty percent of men and 33% of women (26% and 37% for pre‐ and postmenopausal women, respectively) reported having insomnia. The characteristics of participants based on their insomnia status can be found in Table S3. It was observed that individuals who engaged in regular physical activity were less likely to report insomnia. Specifically, among men, 23% of physically active participants reported insomnia compared to 33% of those who were not physically active. Similarly, among women, the rates were 18% for physically active participants and 28% for those who were not physically active. This association was more pronounced among postmenopausal women, where 36% of physically active participants reported insomnia compared to 20% of those who were not physically active. In contrast, among premenopausal women, the difference in insomnia rates between physically active and inactive participants was slightly smaller (20% versus 28%).

With regard to insomnia and sleep duration, more than half of the participants with daily sleep duration of <5 hours reported insomnia (59% and 60% in men and women, respectively) (Table S2). This trend was also more pronounced in postmenopausal women than in premenopausal women with <5 hours of sleep daily (67% versus 35%).

### Association between sleep duration/insomnia and BMD

After excluding participants who lacked sleep information (duration/insomnia), were taking sleep/osteoporosis medication, or had missing variables necessary for each model (as shown in Figure S1), multivariate regression analysis was conducted. The results of the multivariate regression analysis can be found in Table 3. Model 2 was selected due to lower negative AIC value, i.e., a better fit to the data, compared to model 3 in all groups. No association between daily sleep duration and BMD was found in men (model 2: <5 hours; 5 to <7 hours; ≥9 hours was 0.991 [95% CI: 0.946 to 1.039], *p* = 0.720; 0.993 [95% CI: 0.966 to 1.021], *p* = 0.613; 0.971 [95% CI: 0.806 to 1.170], *p* = 0.757, respectively). To interpret this, BMD in participants who slept <5 hours, 5 to <7 hours, and ≥9 hours decreased by 0.9%, 0.7%, and 2.9% compared to participants who slept 7 to <9 hours. However, women who slept 5 to <7 hours daily had significantly higher BMD compared with those who slept 7 to <9 hours daily (1.039 [95% CI: 1.012 to 1.066], *p* = 0.002). This association was observed in subgroups of pre‐ and postmenopausal women (1.058 [95% CI: 1.009 to 1.108], *p* = 0.020; 1.035 [95% CI: 1.002 to 1.068], *p* = 0.037, respectively).

**Table 3:** Association between Sleep Duration/Insomnia and Bone Mineral Density

	Univariate		Model 1		Model 2		Model 3	
	exp(beta) (95% CI)	*p*‐value	exp(beta) (95% CI)	*p*‐value	exp(beta) (95% CI)	*p*‐value	exp(beta) (95% CI)	*p*‐value
Sleep duration, reference 7 to <9 hours^a^								
Men	*N* = 845		*N* = 845		*N* = 842		*N* = 842	
<5 hours	0.997 (0.949 to 1.048)	0.907	0.986 (0.940 to 1.034)	0.565	0.991 (0.946 to 1.039)	0.720	0.991 (0.946 to 1.040)	0.723
5 to <7 hours	1.004 (0.976 to 1.033)	0.776	0.988 (0.961 to 1.016)	0.411	0.993 (0.966 to 1.021)	0.613	0.992 (0.965 to 1.020)	0.561
≥9 hours	0.975 (0.801 to 1.186)	0.797	0.967 (0.802 to 1.166)	0.727	0.971 (0.806 to 1.170)	0.757	0.959 (0.796 to 1.157)	0.665
Women	*N* = 713		*N* = 713		*N* = 712		*N* = 712	
<5 hours	1.020 (0.967 to 1.075)	0.470	1.010 (0.965 to 1.057)	0.677	1.017 (0.972 to 1.064)	0.460	1.018 (0.973 to 1.065)	0.435
5 to <7 hours	1.045 (1.014 to 1.077)	0.005	1.036 (1.009 to 1.063)	0.008	1.039 (1.012 to 1.066)	0.004	1.040 (1.013 to 1.067)	0.004
≥9 hours	0.748 (0.622 to 0.899)	0.002	0.880 (0.750 to 1.033)	0.118	0.898 (0.766 to 1.052)	0.184	0.903 (0.769 to 1.059)	0.209
Premenopausal women	*N* = 259		*N* = 259		*N* = 261		*N* = 261	
<5 hours	1.017 (0.935 to 1.105)	0.699	1.010 (0.930 to 1.095)	0.819	1.016 (0.936 to 1.103)	0.702	1.016 (0.935 to 1.103)	0.715
5 to <7 hours	1.052 (1.003 to 1.103)	0.037	1.053 (1.005 to 1.104)	0.029	1.058 (1.009 to 1.108)	0.020	1.057 (1.008 to 1.108)	0.024
Postmenopausal women	*N* = 414		*N* = 414		*N* = 413		*N* = 413	
<5 hours	1.002 (0.945 to 1.063)	0.946	0.993 (0.938 to 1.050)	0.796	1.006 (0.951 to 1.063)	0.841	1.008 (0.953 to 1.066)	0.777
5 to <7 hours	1.043 (1.008 to 1.079)	0.016	1.032 (0.999 to 1.066)	0.061	1.035 (1.002 to 1.068)	0.037	1.037 (1.004 to 1.071)	0.028
≥9 hours	0.793 (0.653 to 0.964)	0.020	0.902 (0.748 to 1.089)	0.284	0.945 (0.784 to 1.138)	0.550	0.959 (0.795 to 1.156)	0.660
Insomnia, reference not insomnia^b^								
Men	*N* = 845		*N* = 845		*N* = 842		*N* = 842	
Insomnia	0.989 (0.964 to 1.014)	0.381	0.991 (0.967 to 1.016)	0.468	0.994 (0.970 to 1.019)	0.658	0.994 (0.970 to 1.018)	0.610
Women	*N* = 713		*N* = 713		*N* = 712		*N* = 712	
Insomnia	0.986 (0.961 to 1.012)	0.290	0.999 (0.977 to 1.021)	0.937	1.006 (0.984 to 1.028)	0.618	1.006 (0.984 to 1.029)	0.589
Premenopausal women	*N* = 261		*N* = 261		*N* = 261		*N* = 261	
Insomnia	1.017 (0.975 to 1.061)	0.428	1.018 (0.976 to 1.061)	0.410	1.022 (0.980 to 1.066)	0.306	1.023 (0.980 to 1.067)	0.304
Postmenopausal women	*N* = 415		*N* = 415		*N* = 414		*N* = 414	
Insomnia	0.997 (0.969 to 1.025)	0.808	0.993 (0.967 to 1.020)	0.617	1.001 (0.975 to 1.028)	0.959	1.001 (0.975 to 1.028)	0.947

*Note*: Model 1 was adjusted for age, menopausal status, early menopause, and BMI (menopausal status were used for women and early menopause were only used for postmenopausal women); model 2, which was further adjusted for physical activity, smoking, and alcohol intake; and model 3, which was further adjusted for diabetes mellitus and dyslipidemia. The estimated coefficient in exponentiated form is denoted by (exp) beta. This indicates the dependent variable (mean BMD) increases/decreases by one unit in the independent variables based on a comparison of participants in the corresponding group to the participants in the reference group. For example, if (exp)beta is 1.2, then the dependent value in the corresponding group increases by 1.2 or 20% compared to the reference group.

Abbreviations: AIC = Akaike information criterion; BMI = body mass index; CI = confidence interval; exp = exponential.

^a^ AIC for models 2 and 3 were −658 and −655 in men, −794 and −791 in women, −245 and −241 in premenopausal women, and −507 and −505 in premenopausal women, respectively. Large negative values in AIC were considered better fitted model, e.g., model 2 with AIC = −658 was selected for men.

^b^ AIC for Models 1, 2, and 3 were −662 and −659 in men, −793 and −790 in women, −245 and −241 in premenopausal women, −507 and −505 in premenopausal women, respectively. Large negative values in AIC were considered better fitted model, e.g., model 2 with AIC = −662 was selected for men.

Regarding the relationship between insomnia and BMD, no association was found in all groups; insomnia vs not insomnia was 0.994 (95% CI: 0.970 to 1.019, *p* = 0.658) in men and 1.006 (95% CI: 0.984 to 1.028, *p* = 0.618) in women (1.022 [95% CI: 0.980 to 1.066, *p* = 0.306] and 1.001 [95% CI: 0.975 to 1.028, *p* = 0.959] in pre‐ and postmenopausal women, respectively).

When multiple imputation was applied to account for missing values (>5%) in postmenopausal women, the results remained consistent, with the exception that the BMD difference between women who slept 5 to <7 hours daily and those sleeping 7 to <9 house daily was no longer statistically significant (1.027 [95% CI: 0.996 to 1.059], *p* = 0.092) (Table S4).

## Discussion

In this study, we investigated the relationship between sleep duration/insomnia and BMD in participants who underwent BMD testing during their annual health check‐up. It was observed that approximately 80% of the participants slept less than 7 hours daily. However, after adjusting for relevant factors including age, BMI, menopause‐related factors (only for women), physical activity, smoking status, and alcohol intake, shorter sleep duration (<7 hours) was not found to be associated with lower BMD. Additionally, no significant association between insomnia and BMD was detected across all groups.

No significant association between insomnia and BMD was observed in any of the participant groups studied. However, when examination of participant background characteristics revealed that approximately 40% of participants reported sleeping well, while a third of participants met the criteria for insomnia. Additionally, participants who slept less than 5 hours daily had higher BMI compared to those who slept 5 hours or longer. Moreover, individuals who self‐reported experiencing insomnia symptoms were found to be less likely to engage in regular physical activity. This indicates a possible link between insomnia and reduced participation in physical activity.

In Japan, the average daily sleep duration is generally shorter compared to many other countries.^[^ ^29^ ^]^ According to a recent national survey, it was found that less than 20% of middle‐aged men and women (40 to 59 years old) in Japan slept more than 7 hours daily,^[^ ^19^ ^]^ which is the recommended duration in the United States.^[^ ^30^ ^]^ However, our study findings indicate that sleeping less than 7 hours did not have any adverse effects on BMD among women, regardless of menopausal status. Surprisingly, the study revealed that women who slept between 5 and less than 7 hours had significantly higher BMD compared to those who slept between 7 and less than 9 hours. It is important to note that this association was not observed in men.

The findings of past studies on the association between sleep duration and BMD or higher prevalence of osteoporosis have been largely inconsistent. While some studies reported that a shorter sleep duration showed lower BMD compared to the recommended sleep duration,^[^ ^13^, ^14^, ^15^, ^16^, ^21^ ^]^ others reported that a longer sleep duration was associated with lower BMD or higher prevalence of osteoporosis.^[^ ^13^, ^14^, ^19^, ^30^, ^31^, ^32^ ^]^ Meanwhile, several studies reported no association between short or long sleep duration with BMD or osteoporosis.^[^ ^33^, ^34^, ^35^ ^]^ One possible reason for the inconsistency in findings may be due to variations in the study populations, including differences in sex, age groups, and subgroups based on menopausal status. Additionally, variations in outcome variables, such as focusing on osteoporosis or specific BMD values, could have contributed to the inconsistent results. For example, Chen et al. investigated the impact of a daily sleep duration of <7 hours, 7 to 8 hours, and >8 hours on BMD using a large sample in the UK.^[^ ^14^ ^]^ The authors found that men who slept either fewer or more hours had decreased BMD compared to those with the recommended sleep durations. However, no significant association was observed between sleep duration and BMD in women. Swanson et al. investigated a cohort of men aged ≥65 years old who had a daily sleep duration of <6 hours and 7 to 8 hours and reported no significant difference.^[^ ^33^ ^]^ In younger adults, it has been found that shorter daily sleep duration and sleep disturbance decreased bone formation.^[^ ^36^ ^]^ Moreover, evidence suggests that shorter daily sleep duration is associated with increased odds of experiencing fractures.^[^ ^37^, ^38^, ^39^ ^]^ In our study, shorter daily sleep duration (<5 hours, 5 to <7 hours) showed no adverse effect on BMD; however, BMI in those with the shortest sleep duration (<5 hours) was higher across all groups.

Interestingly, Kobayashi et al. found that a longer daily sleep duration (≥8 hours versus 6 hours) was associated with osteoporosis in women aged 50 years and older. In our study, the number of adults who reported sleeping 9 hours or more daily was relatively small. Hence, we might not have been able to detect any potential association between this sleep duration category and the outcomes examined. It is also important to note that we did not analyze the data using a binomial distribution, which would have assessed whether or not participants had osteoporosis. This aspect highlights the need for further research.

Furthermore, most of the studies, including this one, on the association between sleep duration and BMD or osteoporosis were cross‐sectional in nature. Hence, it is difficult to determine the causal relationship between daily sleep duration and BMD or osteoporosis. We further investigated the impact of insomnia on BMD, and no association was found. This result is consistent with that of other studies.^[^ ^14^, ^21^, ^30^ ^]^ Nevertheless, there is evidence that shorter daily sleep duration was strongly associated with obesity.^[^ ^40^ ^]^ More recently, there is evidence suggesting that both very short (≤5 hours) and long (≥9 hours) daily sleep durations are associated with an increased risk of chronic diseases, including cardiovascular diseases, such as coronary artery disease and heart failure.^[^ ^41^ ^]^ Furthermore, a pooled analysis indicated that both short (<7 hours) and long daily sleep durations (>9 hours) are linked to higher mortality rates from cardiovascular diseases.^[^ ^42^ ^]^ Thus, a daily sleep duration of both very short (e.g., <5) and very long (e.g., >9 hours) are not recommended.

One of the strengths of this study is its use of data from annual health check‐ups. These check‐ups encompassed comprehensive information obtained through the administration of a pre‐examination questionnaire. The completeness of the questionnaire responses was ensured by certified nurses, and participants underwent interviews with clinicians to ascertain their health status. The analysis results showed that our data captured the association between BMD and other well‐established risk factors (Tables S4 and S5). Age and smoking status, but not alcohol intake, were significantly associated with lower BMD. We defined an excessive drinker as an individual who consumes two or more drinks per day, following established guidelines. However, it is worth noting that the threshold for defining excessive drinking may vary across different contexts and populations. It is possible that a higher threshold for excessive drinking could have influenced the results. Additionally, we observed that abstainers had lower BMD compared to excessive drinkers, which may have attenuated the effect.^[^ ^8^ ^]^ Nonetheless, considering the available data and the consideration of relevant factors, we believe that our dataset was appropriate for investigating the association between sleep duration/insomnia and BMD.

### Study limitations

First, some risk factors for decreased BMD or osteoporosis were not investigated, such as intake of micronutrients like calcium, vitamin D, and potassium.^[^ ^43^ ^]^

Second, the determination of daily sleep duration in our study was based on a 2‐hour range. A narrower range of assessment could have provided a more precise understanding of the relationship between daily sleep duration and BMD. Future studies could consider using narrower intervals to obtain more detailed data on sleep duration.

Third, our study relied on self‐reported sleep duration, which introduces the possibility of reporting bias and uncertainty regarding the actual sleep duration. However, a study by Swanson et al. comparing self‐reported sleeping hours with objectively measured sleep duration using actigraphy found that individuals with shorter daily sleep durations (<6 hours) tended to overestimate their sleep duration, while those with the recommended daily sleep duration (7 to 8 hours) provided accurate estimates.^[^ ^33^, ^34^ ^]^ This suggests that despite potential limitations, self‐reported sleep duration can still offer valuable insights, especially when examining average sleep patterns within broad categories. Also, in our case, the sample size of those who reported sleeping <7 hours daily was larger than that who slept ≥7 hours.

Finally, our study used BMD values assessed through calcaneal quantitative ultrasound. This technique is commonly used for osteoporosis screening but not for osteoporosis diagnosis.^[^ ^44^, ^45^ ^]^ Therefore, our study focused on examining the association between sleep and BMD rather than directly assessing the relationship between sleep and osteoporosis. By investigating BMD, we aimed to gain insights into the potential influence of sleep on bone health.

## Conclusion

This study evaluated the association between sleep duration, insomnia, and bone health in a Japanese population. Surprisingly, individuals who slept less than 7 hours per day did not exhibit lower BMD. Instead, it was observed that women who slept 7 to less than 9 hours daily had significantly lower BMD compared to those who slept 5 to less than 7 hours daily. No significant association between insomnia and BMD was found. It is worth noting that the average daily sleep duration in Japan, which is shorter compared to other countries, was not independently linked to lower BMD in this study. However, it is important to exercise caution when interpreting these findings, as previous research indicated that both shorter and longer sleep durations may increase the risk of adverse events, including cardiovascular conditions. Further research is needed to better understand the complex relationship between sleep, bone health, and overall well‐being.

## Author Contributions

**Tsutomu Yamazaki:** Conceptualization; investigation; methodology; supervision; writing – review and editing. **Reiko Yamaura:** Conceptualization; formal analysis; investigation; methodology; project administration; writing – original draft; writing – review and editing. **Hideko Kasahara:** Data curation; methodology; supervision; writing – review and editing. **Satoshi Iimuro:** Investigation; methodology; supervision; writing – review and editing.

## Disclosures

There are no conflicts of interest.

## Patient Consent

The requirement for informed consent was waived for research using existing data under the Japanese ethical guidelines.

### Peer Review

The peer review history for this article is available at https://www.webofscience.com/api/gateway/wos/peer-review/10.1002/jbm4.10820.

## Supporting information


**Data S1** Supporting Information.

## Data Availability

The datasets generated and/or analyzed during the current study are not publicly available due to ethical restrictions but are available from the corresponding author on reasonable request.
