# Supplementary material for: The Association between Sleep and Bone Mineral Density: Cross‐Sectional Study Using Health Check‐up Data in a Local Hospital in Japan
Source: JBMR Plus. 2023 Sep 30;7(12):e10820. doi: 10.1002/jbm4.10820 (PMC10731111; doi:10.1002/jbm4.10820)
Supplement: Supplementary file 1 — Data S1 Supporting Information. [file JBM4-7-e10820-s001.docx]

# Supplemental Materials

**Supplemental Figure 1. Flowchart of Participant Selection for Analysis**

**
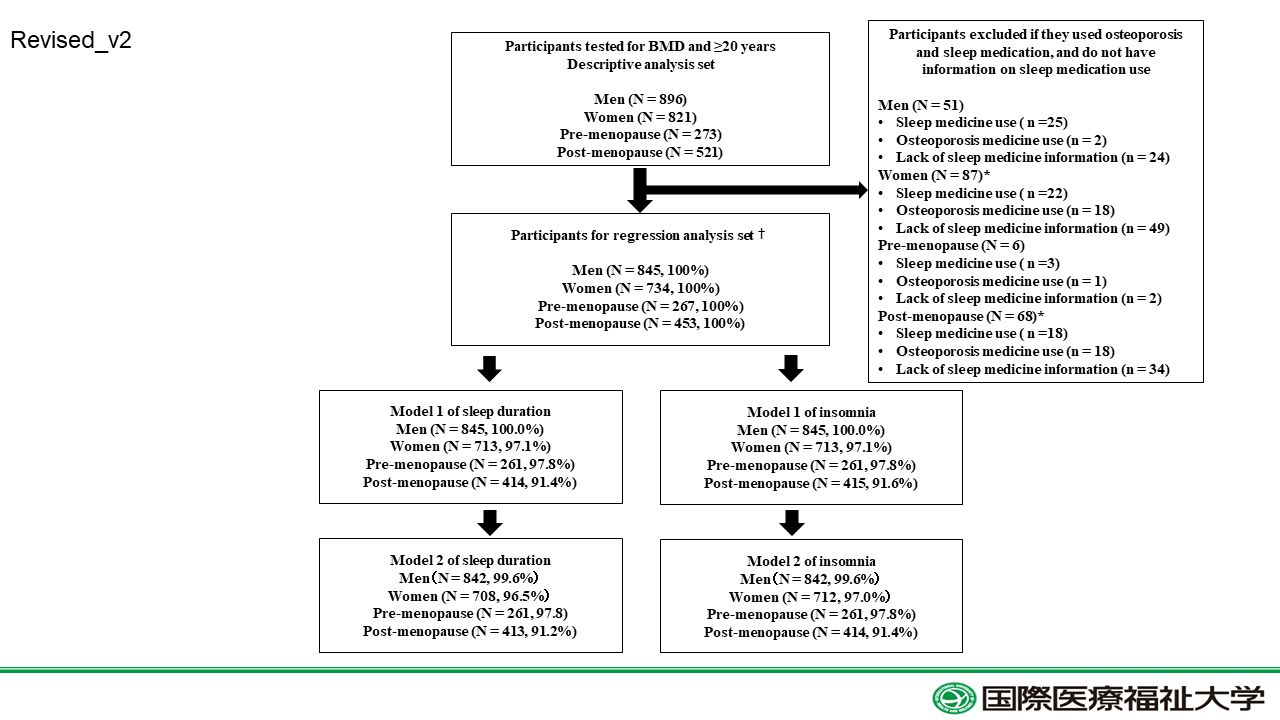
**

*Some participants met multiple exclusion criteria.

†Detailed information on missing variables are tabulated below. Proportion of missing records in post-menopause were only exceeded >5 %.

|  | Men | Women | Pre-menopause | Post-menopause |
| --- | --- | --- | --- | --- |
|  | (N = 845) | (N = 734) | (N = 267) | (N = 453) |
| Age | 0 (0.0%) | 0 (0.0%) | 0 (0.0%) | 0 (0.0%) |
| BMI | 0 (0.0%) | 7 (1%) | 6 (2.2%) | 1 (0.2%) |
| Postmenopausal status | — | 14 (1.9%) | — | — |
| Early menopause | — | — | — | 37 (8.2%) |
| Physically active | 0 (0.0%) | 0 (0.0%) | 0 (0.0%) | 0 (0.0%) |
| Smoking status | 1 (0.1%) | 1 (0.1%) | 0 (0.0%) | 1 (0.2%) |
| Excessive drinker | 2 (0.2%) | 0 (0.0%) | 0 (0.0%) | 0 (0.0%) |
| Dyslipidemia | 0 (0.0%) | 0 (0.0%) | 0 (0.0%) | 0 (0.0%) |
| Diabetes mellitus | 0 (0.0%) | 0 (0.0%) | 0 (0.0%) | 0 (0.0%) |
| Sleep duration | 0 (0.0%) | 4 (0.5%) | 2 (0.7%) | 2 (0.4%) |
| Insomnia | 0 (0.0%) | 0 (0.0%) | 0 (0.0%) | 0 (0.0%) |

**Supplemental Table 1. Pre-examination Health Status Questionnaire**

**If more than one answer is given, check the ones that applies the most to you. Only check the option/s that applied to you. If none applies, you may leave it blank.**

- **Physical activity status and exercise habits**

| **What is your daily activity intensity? (Please answer this question even if you are a housewife)** | **☐ You spend most of the day reading, studying, talking, watching TV or listening to music in a seated or lying position apart from relatively slow walking for about an hour such as walking, shopping, etc. For example, elderly persons at home and housewives, etc.** |
| --- | --- |
|  | **☐** **You do relatively more standing work, such as walking or riding to work or commuting, customer service, and household chores for about two hours daily, but you spend most of your day in a seated position during work. For example, salespersons, sales, drivers, couples with small children.** |
|  | **☐ You spend two hours in a standing position daily which is described in the previous row, and you engage in relatively strenuous physical activities such as fast walking or cycling for about an hour a day. Or you engage in relatively strenuous work such as farming or fishing for about an hour a day, while the majority of your work is in a standing position. For example, agriculture, fishing and construction work, etc.** |
|  | **☐ You are engaged in work such as strenuous training, hauling lumber or farming during the busy farming season for about an hour a day. For example, physical workers.** |
| **What is your work schedule like?** | **☐ Daytime work only** |
|  | **☐ Shift work** |
|  | **☐ Night work only** |
| **Approximately how long do you sleep?** | **☐ <5 hours** |
|  | **☐ 5 to <7 hours** |
|  | **☐ 7 to <9 hours** |
|  | **☐ ≥9 hours** |
| **Do you sleep well (Please select only one answer)** | **☐ Sleep well** |
|  | **☐ Difficulty maintaining sleep** |
|  | **☐ Difficulty initiating sleep** |
|  | **☐ Early-morning awakening** |
|  | **☐ Taking sleep medication** |
| **Do you get enough rest with sleep?** | **☐ Yes** |
| **Do you engage in light sweaty exercise for at least 30 minutes at a time, at least 2 days a week for at least 1 year?** | **☐ Yes** |

- **Smoking and drinking status.**

| **What is your current smoking status?** | **☐** **Currently smoking (have smoked a total of 100 or more cigarettes in your lifetime, or have smoked for at least 6 months, and have smoked for the last month)** |
| --- | --- |
|  | **☐** **Smoked in the past but have quit now (quit smoking)** |
|  | **☐ Never smoked** |
| **How often do you drink alcohol?** | **☐ Everyday** |
|  | **☐ Sometimes** |
|  | **☐ Hardly drink (unable to drink)** |
| **For those who drink alcohol. If 180 mL of sake, 110 mL of 25% shochu, 500 mL of beer, 60 mL of whiskey, and 240 mL of wine are considered one drink, approximately how much alcohol do you drink in one seating?** | **☐ <1 drink** |
|  | **☐ 1 to <2 drinks** |
|  | **☐ 2 to <3 drinks** |
|  | **☐ ≥3 drinks** |

- **Questions only for women**

| **Are you currently premenopausal?** | **☐ Yes** |
| --- | --- |
| **If menopausal, at what age was menopause?** | **years old** |

- **Questions for digestive system examination**

| **Are you using blood glucose-lowering medications or insulin injections?** | **☐ Yes** |
| --- | --- |
| **Are you taking medication to lower cholesterol or triglycerides?** | **☐ Yes** |

- **Medical history. Please fill in only those diseases diagnosed by a physician.**

|  | **Previously treated with medication (treatment history)** | **Currently under regular observation at a specific medical facility (under observation)** | **Currently going to the hospital and being treated with medication (under treatment)** | **Has been indicated in the past but not treated (left untreated)** |
| --- | --- | --- | --- | --- |
| **Diabetes mellitus** | **☐** | **☐** | **☐** | **☐** |
| **Dyslipidemia (cholesterol, triglycerides)** | **☐** | **☐** | **☐** | **☐** |
| **Osteoporosis** | **☐** | **☐** | **☐** | **☐** |

| **Spine (vertebral compression fracture), wrist, or femoral neck fractured to the extent of a fall (excluding traumatic fractures)** | **☐ Yes** |
| --- | --- |

**Supplemental Table 2. Participant Characteristics by Sleep Duration**

**Supplemental Table 2a. Participant Characteristics by Sleep Duration for Men**

| **N = 894** | **<5 hours**  **(N = 73)** | **5 to <7 hours**  **(N = 629)** | **7 to <9 hours**  **(N = 189)** | **≥9 hours**  **(N = 3)** |
| --- | --- | --- | --- | --- |
| Age, years | 54 (48, 63) | 54 (45, 63) | 58 (47, 68) | 68 (48, 70) |
| BMI, kg/m2 | 25.8  (23.1, 28.1) | 24.4  (22.3, 26.7) | 24.0  (21.9, 26.3) | 23.1  (20.5, 26.1) |
| Missing |  |  |  |  |
| Postmenopausal women, n (%) | – | – | – | – |
| Missing |  |  |  |  |
| Early menopause, n (%) | – | – | – | – |
| Missing |  |  |  |  |
| Physically active, n (%) | 17 (23.3%) | 181 (28.8%) | 72 (38.1%) | 0 (0.0%) |
| Smoking status, n (%) |  |  |  |  |
| Current smoker | 27 (37.0%) | 199 (31.7%) | 45 (23.8%) | 1 (33.3%) |
| Past smoker | 31 (42.5%) | 285 (45.4%) | 100 (52.9%) | 0 (0.0%) |
| Never smoker | 15 (20.5%) | 144 (22.9%) | 44 (23.3%) | 2 (66.7%) |
| Missing | 0 | 1 | 0 | 0 |
| Excessive drinker, n (%) | 6 (8.2%) | 64 (10.2%) | 25 (13.2%) | 0 (0.0%) |
| Missing | 0 | 2 | 0 | 0 |
| Bone mineral density, %YAM | 79 (71, 88) | 79 (72, 89) | 79 (71, 89) | 69 (68, 86) |
| Dyslipidemia, n (%) | 47 (64.4%) | 377 (59.9%) | 116 (61.4%) | 1 (33.3%) |
| Diabetes mellitus, n (%) | 14 (19.2%) | 116 (18.4%) | 30 (15.9%) | 2 (66.7%) |
| Sleep satisfaction, n (%) |  |  |  |  |
| Sleep well | 15 (21.7%) | 257 (41.9%) | 89 (47.6%) | 0 (0.0%) |
| Difficulty maintaining sleep | 17 (24.6%) | 189 (30.8%) | 58 (31.0%) | 2 (66.7%) |
| Difficulty in initiating sleep | 17 (24.6%) | 77 (12.6%) | 19 (10.2%) | 1 (33.3%) |
| Early-morning awaking | 9 (13.0%) | 62 (10.1%) | 10 (5.3%) | 0 (0.0%) |
| Taking sleep medication | 6 (8.7%) | 11 (1.8%) | 5 (2.7%) | 0 (0.0%) |
| Multiple answers* | 5 (7.2%) | 17 (2.8%) | 6 (3.2%) | 0 (0.0%) |
| Missing | 4 | 16 | 2 | 0 |
| Not enough rest with sleep, n (%) | 51 (72.9%) | 239 (38.7%) | 35 (18.5%) | 0 (0.0%) |
| Missing | 3 | 11 | 0 | 0 |
| Insomnia, n (%) | 41 (59.4%) | 192 (31.3%) | 28 (15.0%) | 0 (0.0%) |
| Missing | 4 | 16 | 2 | 0 |

*Individuals who did not select “Sleep well” and selected ≥ 2 answers from others were counted as “multiple answers”.

Continuous variables are presented as median in the 1^st^ and 3^rd^ quartiles, and categorical variables are presented as % of the total excluding missing.

BMI = body mass index; YAM = young adult mean

**Supplemental Table 2b. Participant Characteristics by Sleep Duration for Women**

| **N = 809** | **< 5 hours**  **N = 58** | **5 to < 7 hours**  **N = 592** | **7 to < 9 hours**  **N = 156** | **≥ 9 hours**  **N = 3** |
| --- | --- | --- | --- | --- |
| Age, years | 57 (49, 65) | 55 (46, 63) | 57 (45, 65) | 77 (74, 83) |
| BMI, kg/m2 | 23.2  (21.5, 25.8) | 21.9  (19.6, 24.0) | 21.6  (19.6, 24.1) | 17.1  (16.9, 21.7) |
| Missing | 0 | 5 | 2 | 0 |
| Postmenopausal women, n (%) | 37 (68.5%) | 376 (65.3%) | 101 (65.6%) | 3 (100.0%) |
| Missing | 4 | 16 | 2 | 0 |
| Early menopause, n (%) | 5 (9.8%) | 45 (8.2%) | 13 (8.8%) | 0 (0.0%) |
| Missing | 7 | 44 | 9 | 1 |
| Physically active, n (%) | 9 (15.5%) | 149 (25.2%) | 43 (27.6%) | 0 (0.0%) |
| Smoking status, n (%) |  |  |  |  |
| Current smoker | 6 (10.3%) | 48 (8.1%) | 7 (4.5%) | 0 (0.0%) |
| Past smoker | 14 (24.1%) | 118 (20.0%) | 33 (21.2%) | 1 (33.3%) |
| Never smoker | 38 (65.5%) | 425 (71.9%) | 116 (74.4%) | 2 (66.7%) |
| Missing | 0 | 1 | 0 | 0 |
| Excessive drinker, n (%) | 0 (0.0%) | 11 (1.9%) | 3 (1.9%) | 0 (0.0%) |
| Missing | 0 | 1 | 0 | 0 |
| Bone mineral density, %YAM | 73 (67, 85) | 75 (68, 84) | 73 (67, 81) | 54 (54, 56) |
| Dyslipidemia, n (%) | 30 (51.7%) | 285 (48.1%) | 83 (53.2%) | 2 (66.7%) |
| Diabetes mellitus, n (%) | 6 (10.3%) | 39 (6.6%) | 9 (5.8%) | 1 (33.3%) |
| Sleep satisfaction, n (%) |  |  |  |  |
| Sleep well | 16 (28.1%) | 223 (40.0%) | 86 (57.3%) | 0 (0.0%) |
| Difficulty in maintaining sleep | 20 (35.1%) | 219 (39.3%) | 41 (27.3%) | 2 (66.7%) |
| Difficulty in initiating sleep | 11 (19.3%) | 67 (12.0%) | 10 (6.7%) | 1 (33.3%) |
| Early-morning awaking | 5 (8.8%) | 29 (5.2%) | 2 (1.3%) | 0 (0.0%) |
| Taking sleep medication | 1 (1.8%) | 10 (1.8%) | 5 (3.3%) | 0 (0.0%) |
| Multiple answers* | 4 (7.0%) | 9 (1.6%) | 6 (4.0%) | 0 (0.0%) |
| Missing | 1 | 35 | 6 | 0 |
| Not enough rest with sleep, n (%) | 48 (84.2%) | 252 (45%) | 30 (20.0%) | 1 (33.3%) |
| Missing | 1 | 31 | 6 | 0 |
| Insomnia, n (%) | 34 (59.6%) | 197 (35.4%) | 24 (16.0%) | 1 (33.3%) |
| Missing | 1 | 35 | 6 | 0 |

*Individuals who did not select “Sleep well” and selected ≥ 2 answers from others were counted as “multiple answers”.

Continuous variables are presented as median in the 1^st^ and 3^rd^ quartiles, and categorical variables are presented as % of the total excluding missing.

BMI = body mass index; YAM = young adult mean

**Supplemental Table 2c. Participant Characteristics by Sleep Duration for Premenopausal Women**

| **N = 220** | **< 5 hours**  **(N = 17)** | **5 to < 7 hours**  **(N = 200)** | **7 to < 9 hours**  **(N = 53)** | **≥ 9 hours**  **(N = 0)** |
| --- | --- | --- | --- | --- |
| Age, years | 42 (34, 49) | 44 (40, 48) | 43 (39, 45) | – |
| BMI, kg/m2 | 21.8  (19.2, 23.5) | 21.6  (19.6, 23.8) | 20.6  (18.9, 22.7) | – |
| Missing | 0 | 4 | 2 |  |
| Postmenopausal women, n (%) | – | – | – | – |
| Missing |  |  |  |  |
| Early menopause, n (%) | – | – | – | – |
| Missing |  |  |  |  |
| Physically active, n (%) | 1 (5.9%) | 29 (14.5%) | 11 (20.8%) | – |
| Smoking status, n (%) |  |  |  |  |
| Current smoker | 2 (11.8%) | 21 (10.5%) | 1 (1.9%) | – |
| Past smoker | 4 (23.5%) | 42 (21.0%) | 13 (24.5%) | – |
| Never smoker | 11 (64.7%) | 137 (68.5%) | 39 (73.6%) | – |
| Missing | 0 | 0 | 0 |  |
| Excessive drinker, n (%) | 0 (0.0%) | 4 (2.0%) | 1 (1.9%) | – |
| Missing | 0 | 0 | 0 |  |
| Bone mineral density, %YAM | 82 (73, 89) | 82 (75, 92) | 80 (75, 86) | – |
| Dyslipidemia, n (%) | 3 (17.6%) | 55 (27.5%) | 15 (28.3%) | – |
| Diabetes mellitus, n (%) | 0 (0.0%) | 6 (3.0%) | 0 (0.0%) | – |
| Sleep satisfaction, n (%) |  |  |  |  |
| Sleep well | 9 (52.9%) | 90 (45.2%) | 34 (64.2%) | – |
| Difficulty in maintaining sleep | 6 (35.3%) | 73 (36.7%) | 15 (28.3%) | – |
| Difficulty in initiating sleep | 2 (11.8%) | 26 (13.1%) | 1 (1.9%) | – |
| Early-morning awaking | 0 (0.0%) | 8 (4.0%) | 0 (0.0%) | – |
| Taking sleep medication | 0 (0.0%) | 2 (1.0%) | 1 (1.9%) | – |
| Multiple answers* | 0 (0.0%) | 0 (0.0%) | 2 (3.8%) | – |
| Missing | 0 | 1 | 0 |  |
| Not enough rest with sleep, n (%) | 14 (82.4%) | 75 (37.5%) | 11 (20.8%) | – |
| Missing |  |  |  |  |
| Insomnia, n (%) | 6 (35.3%) | 55 (27.6%) | 8 (15.1%) | – |
| Missing | 0 | 1 | 0 |  |

*Individuals who did not select “Sleep well” and selected ≥ 2 answers from others were counted as “multiple answers”.

Continuous variables are presented as median in the 1^st^ and 3^rd^ quartiles, and categorical variables are presented as % of the total excluding missing.

BMI = body mass index; YAM = young adult mean

**Supplemental Table 2d. Participant Characteristics by Sleep Duration for Postmenopausal Women**

| **N = 517** | **< 5 hours**  **(N = 37)** | **5 to < 7 hours**  **(N = 376)** | **7 to < 9 hours**  **(N = 101)** | **≥ 9 hours**  **(N = 3)** |
| --- | --- | --- | --- | --- |
| Age, years | 62 (57, 67) | 60 (55, 67) | 63 (57, 67) | 77 (74, 83) |
| BMI, kg/m2 | 24.1  (21.7, 26.0) | 22.1  (19.8, 24.3) | 22.2  (20.5, 25.3) | 17.1  (16.9, 21.7) |
| Missing | 0 | 1 | 0 | 0 |
| Postmenopausal women, n (%) | – | – | – | – |
| Missing |  |  |  |  |
| Early menopause, n (%) | 5 (14.7%) | 45 (12.9%) | 13 (13.8%) | 0 (0.0%) |
| Missing | 3 | 28 | 7 | 1 |
| Physically active, n (%) | 7 (18.9%) | 117 (31.1%) | 32 (31.7%) | 0 (0.0%) |
| Smoking status, n (%) |  |  |  |  |
| Current smoker | 3 (8.1%) | 25 (6.7%) | 6 (5.9%) | 0 (0.0%) |
| Past smoker | 10 (27.0%) | 73 (19.5%) | 18 (17.8%) | 1 (33.3%) |
| Never smoker | 24 (64.9%) | 277 (73.9%) | 77 (76.2%) | 2 (66.7%) |
| Missing | 0 | 1 | 0 | 0 |
| Excessive drinker, n (%) | 0 (0.0%) | 7 (1.9%) | 2 (2.0%) | 0 (0.0%) |
| Missing | 0 | 1 | 0 | 0 |
| Bone mineral density, %YAM | 70 (65, 76) | 72 (66, 80) | 70 (65, 76) | 54 (54, 56) |
| Dyslipidemia, n (%) | 25 (67.6%) | 228 (60.6%) | 68 (67.3%) | 2 (66.7%) |
| Diabetes mellitus, n (%) | 6 (16.2%) | 32 (8.5%) | 9 (8.9%) | 1 (33.3%) |
| Sleep satisfaction, n (%) |  |  |  |  |
| Sleep well | 7 (19.4%) | 130 (37.2%) | 51 (53.1%) | 0 (0.0%) |
| Difficulty in maintaining sleep | 11 (30.6%) | 141 (40.4%) | 26 (27.1%) | 2 (66.7%) |
| Difficulty in initiating sleep | 8 (22.2%) | 40 (11.5%) | 9 (9.4%) | 1 (33.3%) |
| Early-morning awaking | 5 (13.9%) | 21 (6.0%) | 2 (2.1%) | 0 (0.0%) |
| Taking sleep medication | 1 (2.8%) | 8 (2.3%) | 4 (4.2%) | 0 (0.0%) |
| Multiple answers* | 4 (11.1%) | 9 (2.6%) | 4 (4.2%) | 0 (0.0%) |
| Missing | 1 | 27 | 5 | 0 |
| Not enough rest with sleep, n (%) | 30 (83.3%) | 171 (48.6%) | 19 (19.8%) | 1 (33.3%) |
| Missing | 1 | 24 | 5 | 0 |
| Insomnia, n (%) | 24 (66.7%) | 138 (39.5%) | 16 (16.7%) | 1 (33.3%) |
| Missing | 1 | 27 | 5 | 0 |

*Individuals who did not select “Sleep well” and selected ≥ 2 answers from others were counted as “multiple answers”.

Continuous variables are presented as median in the 1^st^ and 3^rd^ quartiles, and categorical variables are presented as % of the total excluding missing.

BMI = body mass index; YAM = young adult mean

**Supplemental Table 3. Participant Characteristics by Insomnia**

**Supplemental Table 3a. Participant Characteristics by Insomnia of Men and Women**

|  | **Men**  **(N =872)** | | **Women**  **(N = 772)** | |
| --- | --- | --- | --- | --- |
|  | **Not insomnia**  **(N = 611)** | **Insomnia**  **(N = 261)** | **Not insomnia**  **(N = 515)** | **Insomnia**  **(N = 257)** |
| Age, years | 54 (46, 65) | 55 (46, 63) | 55 (45, 64) | 56 (48, 64) |
| BMI, kg/m2 | 24.2   (22.0, 26.4) | 24.9   (22.7, 27.4) | 22.0   (19.7, 24.3) | 21.8   (20.0, 24.4) |
| Missing | 0 | 0 | 5 | 2 |
| Postmenopausal women, n (%) | – | – | 307 (60.3%) | 180 (72.3%) |
| Missing |  |  | 6 | 8 |
| Early menopause, n (%) | – | – | 38 (7.8%) | 19 (8.2%) |
| Missing |  |  | 30 | 24 |
| Physically active, n (%) | 200 (32.7%) | 60 (23.0%) | 144 (28.0%) | 46 (17.9%) |
| Smoking status, n (%) |  |  |  |  |
| Current smoker | 193 (31.6%) | 72 (27.6%) | 37 (7.2%) | 22 (8.6%) |
| Past smoker | 274 (454.9%) | 130 (49.8%) | 96 (18.7%) | 63 (24.5%) |
| Never smoker | 143 (23.4%) | 59 (22.6%) | 381 (74.1%) | 172 (66.9%) |
| Missing | 1 | 0 | 1 | 0 |
| Excessive drinker, n (%) | 63 (10.3%) | 31 (11.9%) | 9 (1.8%) | 5 (1.9%) |
| Missing | 2 | 0 | 1 | 0 |
| Bone mineral density, %YAM | 79 (72, 90) | 79 (71, 88) | 75 (68, 84) | 74 (66, 83) |
| Dyslipidemia, n (%) | 351 (57.4%) | 177 (67.8%) | 243 (47.2%) | 141 (54.9%) |
| Diabetes mellitus, n (%) | 95 (15.5%) | 62 (23.8%) | 34 (6.6%) | 19 (7.4%) |
| Sleep duration, n (%) |  |  |  |  |
| <5 hours | 28 (4.6%) | 41 (15.7%) | 23 (4.5%) | 34 (13.3%) |
| 5 to <7 hours | 421 (68.9%) | 192 (73.6%) | 360 (70.5%) | 197 (77.0%) |
| 7 to <9 hours | 159 (26.0%) | 28 (10.7%) | 126 (24.7%) | 24 (9.4%) |
| ≥9h hours | 3 (0.5%) | 0 (0.0%) | 2 (0.4%) | 1 (0.4%) |
| Missing | 0 | 0 | 4 | 1 |
| Sleep well, n (%) |  |  |  |  |
| Sleep well | 361 (59.1%) | 0 (0.0%) | 327 (63.5%) | 0 (0%.0) |
| Difficulty in maintaining sleep | 157 (25.7%) | 109 (41.8%) | 131 (25.4%) | 152 (59.1%) |
| Difficulty in initiating sleep well | 32 (5.2%) | 82 (31.4%) | 23 (4.5%) | 66 (25.7%) |
| Early-morning awaking | 42 (6.9%) | 39 (14.9%) | 19 (3.7%) | 18 (7.0%) |
| Taking sleep medication | 8 (1.3%) | 14 (5.4%) | 10 (1.9%) | 7 (2.7%) |
| Multiple answers* | 11 (1.8%) | 17 (6.5%) | 5 (1.0%) | 14 (5.4%) |
| Not enough rest with sleep, n (%) | 64 (10.5%) | 261 (100.0%) | 73 (14.2%) | 257 (100.0%) |

*Individuals who did not select “Sleep well” and selected ≥ 2 answers from others were counted as “multiple answers”.

Continuous variables are presented as median in the 1^st^ and 3^rd^ quartiles, and categorical variables are presented as % of the total excluding missing.

BMI = body mass index; YAM = young adult mean

**Supplemental Table 3b. Participant Characteristics by Insomnia of Premenopausal Women and Postmenopausal Women**

|  | **Premenopausal women**  **(N = 271)** | | **Postmenopausal women**  **(N = 487)** | |
| --- | --- | --- | --- | --- |
|  | **Not insomnia**  **(N = 202)** | **Insomnia**  **(N = 69)** | **Not insomnia**  **(N = 307)** | **Insomnia**  **(N = 180)** |
| Age, years | 44 (40, 48) | 43 (39, 47) | 62 (57, 68) | 61 (55, 67) |
| BMI, kg/m2 | 21.6   (19.3, 23.6) | 21.1   (19.9, 23.7) | 22.3   (20.3, 24.8) | 22.2   (20.4, 24.6) |
| Missing | 5 | 1 | 0 | 1 |
| Postmenopausal women, n (%) | – | – | – | – |
| Missing |  |  |  |  |
| Early menopause, n (%) | – | – | 38 (13%) | 19 (11.6%) |
| Missing |  |  | 24 | 16 |
| Physically active, n (%) | 31 (15.3%) | 9 (13.0%) | 111 (36.2%) | 36 (20.0%) |
| Smoking status, n (%) |  |  |  |  |
| Current smoker | 14 (6.9%) | 10 (14.5%) | 21 (6.9%) | 11 (6.1%) |
| Past smoker | 42 (20.8%) | 18 (26.1%) | 53 (17.3%) | 43 (23.9%) |
| Never smoker | 146 (72.3%) | 41 (59.4%) | 232 (75.8%) | 126 (70.0%) |
| Missing |  |  | 1 | 0 |
| Excessive drinker, n (%) | 3 (1.5%) | 2 (2.9%) | 6 (2.0%) | 3 (1.7%) |
| Missing |  |  | 1 | 0 |
| Bone mineral density, %YAM | 81 (75, 91) | 82 (75, 93) | 72 (65, 79) | 71 (65, 79) |
| Dyslipidemia, n (%) | 53 (26.2%) | 22 (31.9%) | 189 (61.6%) | 116 (64.4%) |
| Diabetes mellitus, n (%) | 4 (2.0%) | 2 (2.9%) | 29 (9.4%) | 17 (9.4%) |
| Sleep duration, n (%) |  |  |  |  |
| <5 hours | 11 (5.5%) | 6 (8.7%) | 12 (3.9%) | 24 (13.4%) |
| 5 to <7 hours | 144 (72.0%) | 55 (80%) | 211 (69%) | 138 (77.1%) |
| 7 to <9 hours | 45 (22.5%) | 8 (12%) | 80 (26%) | 16 (8.9%) |
| ≥9h hours | 0 (0.0%) | 0 (0%) | 2 (0.7%) | 1 (0.6%) |
| Missing | 2 | 0 | 2 | 1 |
| Sleep well, n (%) |  |  |  |  |
| Sleep well | 134 (66.3%) | 0 (0.0%) | 189 (61.6%) | 0 (0.0%) |
| Difficulty in maintaining sleep | 48 (23.8%) | 47 (68.1%) | 81 (26.4%) | 99 (55.0%) |
| Difficulty in initiating sleep | 14 (6.9%) | 15 (21.7%) | 9 (2.9%) | 49 (27.2%) |
| Early-morning awaking | 4 (2.0%) | 4 (5.8%) | 15 (4.9%) | 14 (7.8%) |
| Taking sleep medication | 2 (1.0%) | 1 (1.4%) | 8 (2.6%) | 6 (3.3%) |
| Multiple answers* | 0 (0.0%) | 2 (2.9%) | 5 (1.6%) | 12 (6.7%) |
| Not enough rest with sleep, n (%) | 32 (15.8%) | 69 (100.0%) | 39 (12.7%) | 180 (100.0%) |

*Individuals who did not select “Sleep well” and selected ≥ 2 answers from others were counted as “multiple answers”.

Continuous variables are presented for median with 1^st^ and 3^rd^ quartiles, and categorical variables are presented for % to the total excluding missing.

BMI = body mass index; YAM = young adult mean.

**Supplemental Table 4. Regression Model 2 and 3 With Multiple Imputation for Impact of Sleep Duration and Insomnia on Bone Mineral Density for Postmenopausal Women**

|  | **Model 2** |  | **Model 3** |  |
| --- | --- | --- | --- | --- |
|  | **exp(beta) (95% CI)** | **p-value** | **exp(beta) (95% CI)** | **p-value** |
| **Sleep duration, reference 7 to < 9 hours** |  |  |  |  |
| <5 hours | 1.020 (0.967–1.075) | 0.474 | 1.021 (0.968–1.077) | 0.436 |
| 5 to <7 hours | 1.027 (0.996–1.059) | 0.092 | 1.028 (0.997–1.060) | 0.081 |
| ≥9 hours | 0.907 (0.779–1.056) | 0.210 | 0.913 (0.784–1.064) | 0.245 |
| Age | 0.993 (0.991–0.994) | <0.001 | 0.993 (0.991–0.994) | <0.001 |
| BMI | 1.005 (1.001–1.008) | 0.005 | 1.005 (1.002–1.008) | 0.004 |
| Early menopause, reference not early menopause | 0.994 (0.957–1.033) | 0.761 | 0.994 (0.957–1.033) | 0.763 |
| Active, reference not active | 1.024 (0.997–1.051) | 0.087 | 1.025 (0.998–1.053) | 0.074 |
| Smoking status, reference never smoker |  |  |  |  |
| Current smoker | 0.922 (0.877–0.969) | 0.002 | 0.922 (0.877–0.969) | 0.002 |
| Past smoker | 0.95 (0.921–0.981) | 0.002 | 0.949 (0.919–0.98) | 0.001 |
| Excessive drinking, reference no excessive drinking | 1.066 (0.971–1.171) | 0.181 | 1.069 (0.973–1.174) | 0.165 |
| Dyslipidemia, reference not dyslipidemia |  |  | 1.004 (0.978–1.031) | 0.766 |
| Diabetes mellitus, reference not diabetes mellitus |  |  | 0.974 (0.933–1.016) | 0.225 |
|  |  |  |  |  |
| **Insomnia, reference not insomnia** | 0.997 (0.972–1.023) | 0.837 | 0.998 (0.973–1.024) | 0.884 |
| Age | 0.992 (0.991–0.994) | <0.001 | 0.992 (0.991–0.994) | <0.001 |
| BMI | 1.005 (1.002–1.008) | 0.004 | 1.005 (1.002–1.008) | 0.003 |
| Early menopause, reference not early menopause | 0.994 (0.956–1.033) | 0.744 | 0.994 (0.956–1.033) | 0.742 |
| Active, reference not active | 1.025 (0.998–1.053) | 0.075 | 1.026 (0.999–1.054) | 0.063 |
| Smoking status, reference never smoker |  |  |  |  |
| Current smoker | 0.921 (0.876–0.969) | 0.002 | 0.921 (0.876–0.969) | 0.002 |
| Past smoker | 0.951 (0.921–0.982) | 0.002 | 0.950 (0.920–0.98) | 0.002 |
| Excessive drinking, reference no excessive drinking | 1.066 (0.970–1.171) | 0.183 | 1.069 (0.972–1.174) | 0.169 |
| Dyslipidemia, reference not dyslipidemia |  |  | 1.003 (0.977–1.029) | 0.835 |
| Diabetes mellitus, reference not diabetes mellitus |  |  | 0.974 (0.933–1.016) | 0.224 |

Model 2 adjusted for age, early menopause, BMI, physical activity, smoking, and alcohol intake; and model 3 which was further adjusted for diabetes mellitus and dyslipidemia. (exp)beta denotes the estimated coefficient in exponentiated form. This indicates the dependent variable (mean BMD) increases/decreases by one unit in the independent variables comparing participants in the corresponding group to the participants in the reference group. e.g., if (exp)beta is 1.2, then the dependent value in the corresponding group increases by 1.2 or 20% compared to the reference group.

BMI = body mass index; exp = exponential; CI = confidence interval.

**Supplemental Table 5. Regression Model 2 and 3 for Impact of Sleep Duration on Bone Mineral Density**

**Supplemental Table 5a. Regression Model 2 and 3 for Impact of Sleep Duration on Bone Mineral Density for Men (N = 842) and Women (N = 712)**

|  | **Model 2** |  | **Model 3** |  |
| --- | --- | --- | --- | --- |
|  | **exp(beta) (95% CI)** | **p-value** | **exp(beta) (95% CI)** | **p-value** |
| **Men** |  |  |  |  |
| Sleep duration, reference 7 to < 9 hours |  |  |  |  |
| <5 hours | 0.991 (0.946–1.039) | 0.720 | 0.991 (0.946–1.040) | 0.723 |
| 5 to <7 hours | 0.993 (0.966–1.021) | 0.613 | 0.992 (0.965–1.020) | 0.561 |
| ≥9 hours | 0.971 (0.806–1.170) | 0.757 | 0.959 (0.796–1.157) | 0.665 |
| Age | 0.996 (0.995–0.997) | <0.001 | 0.995 (0.994–0.997) | <0.001 |
| BMI | 0.998 (0.995–1.001) | 0.211 | 0.998 (0.995–1.001) | 0.197 |
| Physically active, reference not active | 1.044 (1.018–1.070) | <0.001 | 1.044 (1.018–1.070) | <0.001 |
| Smoking status, reference never smoker |  |  |  |  |
| Current smoker | 0.963 (0.934–0.993) | 0.015 | 0.962 (0.933–0.992) | 0.014 |
| Past smoker | 0.977 (0.949–1.005) | 0.112 | 0.977 (0.949–1.006) | 0.113 |
| Excessive drinking, reference no excessive drinking | 0.978 (0.944–1.013) | 0.221 | 0.979 (0.945–1.015) | 0.253 |
| Dyslipidemia, reference not dyslipidemia |  |  | 0.994 (0.971–1.018) | 0.640 |
| Diabetes mellitus, reference not diabetes mellitus |  |  | 1.020 (0.989–1.052) | 0.205 |
| **Women** |  |  |  |  |
| Sleep duration, reference 7 to < 9 hours |  |  |  |  |
| <5 hours | 1.017 (0.972–1.064) | 0.460 | 1.018 (0.973–1.065) | 0.435 |
| 5 to <7 hours | 1.039 (1.012–1.066) | 0.004 | 1.040 (1.013–1.067) | 0.004 |
| ≥9 hours | 0.898 (0.766–1.052) | 0.184 | 0.903 (0.769–1.059) | 0.209 |
| Age | 0.994 (0.992–0.995) | <0.001 | 0.994 (0.992–0.995) | <0.001 |
| BMI | 1.004 (1.001–1.007) | 0.002 | 1.004 (1.002–1.007) | 0.002 |
| Post menopause, reference pre menopause | 0.972 (0.941–1.004) | 0.086 | 0.972 (0.940–1.004) | 0.082 |
| Physically active, reference not active | 1.029 (1.004–1.054) | 0.021 | 1.030 (1.005–1.055) | 0.018 |
| Smoking status, reference never smoker |  |  |  |  |
| Current smoker | 0.949 (0.912–0.987) | 0.010 | 0.948 (0.911–0.987) | 0.009 |
| Past smoker | 0.969 (0.945–0.995) | 0.018 | 0.969 (0.944–0.994) | 0.016 |
| Excessive drinking, reference no excessive drinking | 1.012 (0.938–1.093) | 0.751 | 1.013 (0.939–1.094) | 0.732 |
| Dyslipidemia, reference not dyslipidemia |  |  | 0.980 (0.940–1.021) | 0.335 |
| Diabetes mellitus, reference not diabetes mellitus |  |  | 1.003 (0.980–1.026) | 0.825 |

Model 2 adjusted for age, menopausal status, early menopause, BMI (menopausal status were used for women and early menopause were only used for postmenopausal women), physical activity, smoking, and alcohol intake; and model 3 which was further adjusted for diabetes mellitus and dyslipidemia. (exp)beta denotes the estimated coefficient in exponentiated form. This indicates the dependent variable (mean BMD) increases/decreases by one unit in the independent variables comparing participants in the corresponding group to the participants in the reference group. e.g., if (exp)beta is 1.2, then the dependent value in the corresponding group increases by 1.2 or 20% compared to the reference group.

BMI = body mass index; exp = exponential; CI = confidence interval.

**Supplemental Table 5b. Regression Model 2 and 3 for Impact of Sleep duration on Bone Mineral Density for Premenopausal Women (N = 261) and Postmenopausal Women (N = 413)**

|  | **Model 2** |  | **Model 3** |  |
| --- | --- | --- | --- | --- |
|  | **exp(beta) (95% CI)** | **p-value** | **exp(beta) (95% CI)** | **p-value** |
| **Premenopausal women** |  |  |  |  |
| Sleep duration, reference 7 to < 9 hours |  |  |  |  |
| <5 hours | 1.016 (0.936–1.103) | 0.702 | 1.016 (0.935–1.103) | 0.715 |
| 5 to <7 hours | 1.058 (1.009–1.108) | 0.020 | 1.057 (1.008–1.108) | 0.024 |
| Age | 0.995 (0.992–0.998) | <0.001 | 0.995 (0.992–0.998) | <0.001 |
| BMI | 1.002 (0.997–1.007) | 0.419 | 1.002 (0.997–1.007) | 0.460 |
| Active, reference not active | 1.038 (0.986–1.094) | 0.156 | 1.038 (0.985–1.094) | 0.164 |
| Smoking status, reference never smoker |  |  |  |  |
| Current smoker | 0.976 (0.913–1.043) | 0.468 | 0.977 (0.913–1.045) | 0.495 |
| Past smoker | 0.988 (0.945–1.034) | 0.608 | 0.987 (0.943–1.034) | 0.580 |
| Excessive drinking, reference no excessive drinking | 0.950 (0.833–1.084) | 0.448 | 0.950 (0.832–1.084) | 0.448 |
| Dyslipidemia, reference not dyslipidemia |  |  | 1.022 (0.902–1.158) | 0.730 |
| Diabetes mellitus, reference not diabetes mellitus |  |  | 0.995 (0.952–1.040) | 0.813 |
| **Postmenopausal women** |  |  |  |  |
| Sleep duration, reference 7 to < 9 hours |  |  |  |  |
| <5 hours | 1.006 (0.951–1.063) | 0.841 | 1.008 (0.953–1.066) | 0.777 |
| 5 to <7 hours | 1.035 (1.002–1.068) | 0.037 | 1.037 (1.004–1.071) | 0.028 |
| ≥9 hours | 0.945 (0.784–1.138) | 0.550 | 0.959 (0.795–1.156) | 0.660 |
| Age | 0.993 (0.991–0.995) | <0.001 | 0.993 (0.991–0.995) | <0.001 |
| BMI | 1.004 (1.001–1.008) | 0.009 | 1.005 (1.001–1.008) | 0.010 |
| Early menopause, reference pre menopause | 0.992 (0.955–1.031) | 0.697 | 0.993 (0.956–1.033) | 0.741 |
| Active, reference not active | 1.026 (0.998–1.054) | 0.073 | 1.026 (0.998–1.055) | 0.070 |
| Smoking status, reference never smoker |  |  |  |  |
| Current smoker | 0.921 (0.874–0.971) | 0.003 | 0.922 (0.874–0.972) | 0.003 |
| Past smoker | 0.952 (0.921–0.984) | 0.004 | 0.951 (0.919–0.983) | 0.003 |
| Excessive drinking, reference no excessive drinking | 1.064 (0.969–1.169) | 0.193 | 1.067 (0.971–1.172) | 0.178 |
| Dyslipidemia, reference not dyslipidemia |  |  | 0.973 (0.932–1.016) | 0.221 |
| Diabetes mellitus, reference not diabetes mellitus |  |  | 1.014 (0.986–1.042) | 0.333 |

Model 2 adjusted for age, menopausal status, early menopause, BMI (menopausal status were used for women and early menopause were only used for postmenopausal women), physical activity, smoking, and alcohol intake; and model 3 which was further adjusted for diabetes mellitus and dyslipidemia. (exp)beta denotes the estimated coefficient in exponentiated form. This indicates the dependent variable (mean BMD) increases/decreases by one unit in the independent variables comparing participants in the corresponding group to the participants in the reference group. e.g., if (exp)beta is 1.2, then the dependent value in the corresponding group increases by 1.2 or 20% compared to the reference group.

BMI = body mass index; exp = exponential; CI = confidence interval.

**Supplemental Table 6. Regression Model 2 and 3 for Impact of Insomnia on Bone Mineral Density**

**Supplemental Table 6a. Regression Model 2 and 3 for Impact of Insomnia on Bone Mineral Density for Men (N = 842) and Women (N = 712)**

|  | **Model 2** |  | **Model 3** |  |
| --- | --- | --- | --- | --- |
|  | **exp(beta) (95% CI)** | **p-value** | **exp(beta) (95% CI)** | **p-value** |
| **Men** |  |  |  |  |
| Insomnia, reference not insomnia | 0.994 (0.970–1.019) | 0.658 | 0.994 (0.970–1.018) | 0.610 |
| Age | 0.996 (0.995–0.997) | <0.001 | 0.996 (0.994–0.997) | <0.001 |
| BMI | 0.998 (0.995–1.001) | 0.214 | 0.998 (0.995–1.001) | 0.199 |
| Active, reference not active | 1.044 (1.018–1.070) | <0.001 | 1.044 (1.018–1.070) | <0.001 |
| Smoking status, reference never smoker |  |  |  |  |
| Current smoker | 0.962 (0.933–0.992) | 0.014 | 0.962 (0.933–0.992) | 0.013 |
| Past smoker | 0.977 (0.949–1.006) | 0.115 | 0.977 (0.949–1.006) | 0.118 |
| Excessive drinking, reference no excessive drinking | 0.979 (0.945–1.014) | 0.237 | 0.980 (0.946–1.016) | 0.272 |
| Dyslipidemia, reference not dyslipidemia |  |  | 0.995 (0.971–1.019) | 0.675 |
| Diabetes mellitus, reference not diabetes mellitus |  |  | 1.020 (0.989–1.052) | 0.212 |
| **Women** |  |  |  |  |
| Insomnia, reference not insomnia | 1.006 (0.984–1.028) | 0.618 | 1.006 (0.984–1.029) | 0.589 |
| Age | 0.993 (0.992–0.995) | <0.001 | 0.993 (0.992–0.995) | <0.001 |
| BMI | 1.004 (1.002–1.007) | 0.002 | 1.005 (1.002–1.007) | 0.001 |
| Post menopause, reference pre menopause | 0.975 (0.944–1.007) | 0.125 | 0.975 (0.943–1.007) | 0.122 |
| Active, reference not active | 1.031 (1.006–1.056) | 0.015 | 1.032 (1.007–1.058) | 0.013 |
| Smoking status, reference never smoker |  |  |  |  |
| Current smoker | 0.950 (0.913–0.989) | 0.012 | 0.949 (0.912–0.988) | 0.011 |
| Past smoker | 0.970 (0.945–0.995) | 0.019 | 0.969 (0.944–0.994) | 0.017 |
| Excessive drinking, reference no excessive drinking | 1.013 (0.938–1.093) | 0.748 | 1.014 (0.939–1.094) | 0.728 |
| Dyslipidemia, reference not dyslipidemia |  |  | 0.979 (0.939–1.021) | 0.316 |
| Diabetes mellitus, reference not diabetes mellitus |  |  | 1.000 (0.978–1.023) | 0.969 |

Model 2 adjusted for age, menopausal status, early menopause, BMI (menopausal status were used for women and early menopause were only used for postmenopausal women), physical activity, smoking, and alcohol intake; and model 3 which was further adjusted for diabetes mellitus and dyslipidemia. (exp)beta denotes the estimated coefficient in exponentiated form. This indicates the dependent variable (mean BMD) increases/decreases by one unit in the independent variables comparing participants in the corresponding group to the participants in the reference group. e.g., if (exp)beta is 1.2, then the dependent value in the corresponding group increases by 1.2 or 20% compared to the reference group.

BMI = body mass index; exp = exponential; CI = confidence interval.

**Supplemental Table 6b. Regression Model 2 and 3 for Impact of Insomnia on Bone Mineral Density for Premenopausal Women (N = 261) and Postmenopausal Women (414)**

|  | **Model 2** |  | **Model 3** |  |
| --- | --- | --- | --- | --- |
|  | **exp(beta) (95% CI)** | **p-value** | **(exp)beta (95% CI)** | **p-value** |
| **Premenopausal women** |  |  |  |  |
| Insomnia, reference not insomnia | 1.022 (0.980–1.066) | 0.306 | 1.023 (0.980–1.067) | 0.304 |
| Age | 0.995 (0.992–0.998) | <0.001 | 0.995 (0.993–0.998) | <0.001 |
| BMI | 1.003 (0.998–1.008) | 0.298 | 1.003 (0.997–1.008) | 0.341 |
| Active, reference not active | 1.036 (0.984–1.091) | 0.180 | 1.036 (0.983–1.092) | 0.186 |
| Smoking status, reference never smoker |  |  |  |  |
| Current smoker | 0.977 (0.913–1.045) | 0.500 | 0.979 (0.914–1.047) | 0.534 |
| Past smoker | 0.988 (0.944–1.034) | 0.600 | 0.986 (0.942–1.033) | 0.555 |
| Excessive drinking, reference no excessive drinking | 0.949 (0.832–1.084) | 0.442 | 0.949 (0.831–1.084) | 0.439 |
| Dyslipidemia, reference not dyslipidemia |  |  | 1.032 (0.911–1.169) | 0.623 |
| Diabetes mellitus, reference not diabetes mellitus |  |  | 0.992 (0.949–1.036) | 0.707 |
| **Postmenopausal women** |  |  |  |  |
| Insomnia, reference not insomnia | 1.001 (0.975–1.028) | 0.959 | 1.001 (0.975–1.028) | 0.947 |
| Age | 0.992 (0.990–0.994) | <0.001 | 0.992 (0.990–0.994) | <0.001 |
| BMI | 1.004 (1.001–1.007) | 0.014 | 1.004 (1.001–1.008) | 0.016 |
| Early menopause, reference not early menopause | 0.991 (0.954–1.031) | 0.665 | 0.992 (0.954–1.032) | 0.693 |
| Active, reference not active | 1.027 (0.999–1.056) | 0.063 | 1.028 (0.999–1.057) | 0.060 |
| Smoking status, reference never smoker |  |  |  |  |
| Current smoker | 0.920 (0.872–0.970) | 0.002 | 0.920 (0.872–0.970) | 0.002 |
| Past smoker | 0.951 (0.920–0.983) | 0.003 | 0.950 (0.919–0.982) | 0.003 |
| Excessive drinking, reference no excessive drinking | 1.065 (0.970–1.170) | 0.188 | 1.067 (0.971–1.172) | 0.176 |
| Dyslipidemia, reference not dyslipidemia |  |  | 0.973 (0.932–1.016) | 0.223 |
| Diabetes mellitus, reference not diabetes mellitus |  |  | 1.011 (0.984–1.039) | 0.432 |

Model 2 adjusted for age, menopausal status, early menopause, BMI (menopausal status were used for women and early menopause were only used for postmenopausal women), physical activity, smoking, and alcohol intake; and model 3 which was further adjusted for diabetes mellitus and dyslipidemia. (exp)beta denotes the estimated coefficient in exponentiated form. This indicates the dependent variable (mean BMD) increases/decreases by one unit in the independent variables comparing participants in the corresponding group to the participants in the reference group. e.g., if (exp)beta is 1.2, then the dependent value in the corresponding group increases by 1.2 or 20% compared to the reference group.

BMI = body mass index; exp = exponential; CI = confidence interval.
